# A multi-stage process including transient polyploidization and EMT precedes the emergence of chemoresistent ovarian carcinoma cells with a dedifferentiated and pro-inflammatory secretory phenotype

**DOI:** 10.18632/oncotarget.5552

**Published:** 2015-10-19

**Authors:** Verena Rohnalter, Katrin Roth, Florian Finkernagel, Till Adhikary, Julia Obert, Kristina Dorzweiler, Maike Bensberg, Sabine Müller-Brüsselbach, Rolf Müller

**Affiliations:** ^1^ Institute of Molecular Biology and Tumor Research (IMT), Center for Tumor Biology and Immunology, Philipps University, Marburg 35043, Germany; ^2^ Cell Imaging Core Facility, Center for Tumor Biology and Immunology, Philipps University, Marburg 35043, Germany

**Keywords:** chemoresistance, giant cancer cells, polyploidy, senescence-associated secretory phenotype (SASP), epithelial-mesenchymal transition (EMT)

## Abstract

DNA-damaging drugs induce a plethora of molecular and cellular alterations in tumor cells, but their interrelationship is largely obscure. Here, we show that carboplatin treatment of human ovarian carcinoma SKOV3 cells triggers an ordered sequence of events, which precedes the emergence of mitotic chemoresistant cells. The initial phase of cell death after initiation of carboplatin treatment is followed around day 14 by the emergence of a mixed cell population consisting of cycling, cell cycle-arrested and senescent cells. At this stage, giant cells make up >80% of the cell population, p21 *(CDKN1A)* in strongly induced, and cell numbers remain nearly static. Subsequently, cell death decreases, p21 expression drops to a low level and cell divisions increase, including regular mitoses of giant cells and depolyploidization by multi-daughter divisions. These events are accompanied by the upregulation of stemness markers and a pro-inflammatory secretory phenotype, peaking after approximately 14 days of treatment. At the same time the cells initiate epithelial to mesenchymal transition, which over the subsequent weeks continuously increases, concomitantly with the emergence of highly proliferative, migratory, dedifferentiated, pro-inflammatory and chemoresistant cells (SKOV3-R). These cells are anchorage-independent and grow in a 3D collagen matrix, while cells on day 14 do not survive under these conditions, indicating that SKOV3-R cells were generated thereafter by the multi-stage process described above. This process was essentially recapitulated with the ovarian carcinoma cell line IGROV-1. Our observations suggest that transitory cells characterized by polyploidy, features of stemness and a pro-inflammatory secretory phenotype contribute to the acquisition of chemoresistance.

## INTRODUCTION

Ovarian cancer ranks fifth as the cause of death from cancer in women with >20,000 new cases annually in the United States, >40,000 in the European Union and >50% of new cases diagnosed worldwide in developing countries [1]. Ovarian cancer has a dire prognosis with an overall 5-year survival rate of <25%. At least 90% of malignant ovarian tumors are carcinomas, presumably originating from the Müllerian epithelium of the ovarian surface and the Fallopian tube, the latter especially in the context of hereditary ovarian cancer [2]. The WHO classification distinguishes serous, mucinous, endometrioid, clear cell, transitional cell and squamous carcinoma [1]. Of these, serous ovarian carcinoma is the most common ovarian cancer, but tumors of other subtypes carry a similarly poor prognosis. For example, the survival of advanced clear cell carcinoma is extremely low due to its insensitivity to platinum-based chemotherapy compared to non-clear cell ovarian cancers [3].

Distinct mutation patterns and expression profiles indicate that high-grade and low-grade serous ovarian cancers are two distinct disease entities, with the latter bearing considerable molecular resemblance with low-grade endometrioid, mucinous and clear-cell carcinomas [1, 2, 4]. These tumor subtypes are collectively referred to as type 1 cancers and typically harbor mutations in components of receptor tyrosine kinase signaling pathways (ERBB2, KRAS, BRAF), phosphatidylinositol-4,5-bisphosphate 3-kinase (PIK3CA), the phosphatidylinositol-3,4,5-trisphosphate phosphatase PTEN, β-catenin (CTNNB1) and the SNI/SNF-related chromatin remodeler ARID1A, they are relatively genetically stable and may arise from benign precursor lesions. In contrast, the aggressive type 2 cancers, including high-grade serous adenocarcinoma, are characterized by frequent TP53 mutations (up to 97%), the occurrence of BRCA1/2 alterations (~20%) and the lack of a clear precursor lesion.

Several features contribute to the fatal nature of ovarian cancer. Tumor cells are usually shed at a very early stage of the disease, and their spread to other pelvic and peritoneal organs is facilitated by the peritoneal fluid serving as a carrier [4]. This passive transcoelemic dissemination represents a primary route to the formation of metastatic lesions. The peritoneal environment, which is formed by a malignant effusion building up in the peritoneal cavity, may be an essential determinant of disease progression. This malignancy-associated ascites is rich in tumor-promoting soluble factors [5], highly tumorigenic cancer cells [6] and different types of immune cells, including large numbers of pro-tumorigenic macrophages (TAMs) [7, 8], supporting tumor cell proliferation, angiogenesis and immune evasion.

Ovarian carcinoma growth and progression are thought to be fueled by cells expressing stem cell markers [9–11] and are therefore referred to as cancer stem cells or cancer propagating cells (CPCs). These are characterized by a low proliferation rate, a high apoptotic threshold and a high capacity for drug elimination, rendering these cells highly chemoresistant. CPCs also have a high tumorigenic potential, which facilitates the formation of metastases. Surface marker expression has been used extensively to identify ovarian CPCs by flow cytometry as cells with high levels of CD44, CD117 (KIT), CD133 (PROM1) and aldehyde dehydrogenase 1 (ALDH1) expression, high intracellular ALDH activity and/or enhanced Hoechst dye extrusion by ABC transporters (side population cells) [12, 13]. However, ovarian cancer cell subpopulations have been isolated that express stemness markers at highly variable levels, in different combinations and with none of these markers being obligatory [9–11, 14, 15], suggesting that a common ovarian CPCs may not exist or has not been identified. Expression of several of these markers has also been associated with a poor clinical outcome, supporting a role for these cells in tumor progression and drug resistance. Thus, even though the cancer stem cell hypothesis is a matter of debate, it is likely that the aberrant expression of stemness-associated genes is an important determinant of tumor progression and recurrence.

CPCs have been proposed to reactivate developmental programs that are used by embryonal stem cells to maintain pluripotency [16], notably the core transcriptional circuitry governed by OCT4, NANOG and SOX2 (core module) [17]. Consistent with this idea OCT4, NANOG and SOX2 are expressed in ovarian CPCs [6, 10, 18–22], and the enforced expression of OCT4 or NANOG enhances the malignancy of ovarian carcinoma cell lines [21]. The expression of pluripotency factors has also been linked to chemoresistance of ovarian cancer cells [18, 23–25], but the underlying mechanisms are poorly understood.

The acquisition of stemness is closely linked to epithelial-mesenchymal transition (EMT) [26–30], a developmental program that transiently disrupts homotypic epithelial cell-cell adhesion and converts epithelial cells to migratory, invasive mesenchymal cells [31]. Cancer EMT, which recapitulates developmental EMT, is the driving force of metastasis. CPC-like cells from ovarian carcinoma patients have a mesenchymal phenotype [32], supporting the view that these cells initiate metastasis. EMT has also been associated with chemoresistance [33–38], emphasizing its relevance with respect to recurrence of the disease.

DNA and spindle damaging agents, such as carboplatin (CPT) and paclitaxel used for the treatment of ovarian cancer, induce polyploidy [39], in particular in the absence of functional p53 [40]. A fraction of these polyploid cells shows the hallmarks of replicative senescence, including cell cycle arrest and high acidic β-galactosidase activity [41]. Surprisingly, these cells are endowed with the potential to regain a para-diploid, highly proliferative state [40, 42]. This reversible polyploidy is associated with the induction of pluripotency markers in lymphoma cell lines, thus linking apparently opposing processes, i.e. stemness and senescence [41]. A similar phenomenon has recently been described for different carcinoma cell lines treated with CoCl_2_ as a hypoxia-mimetic [43].

Chemotherapeutic drugs and radiation are known to trigger processes associated with malignancy and tumor progression, including senescence [44, 45], a pro-inflammatory senescence-associated secretory phenotype (SASP) [46], polyploidization and giant cell formation [40, 42, 47, 48], expression of stemness-related markers [49] and EMT [50, 51]. Collectively, these observations suggest that the acquisition of a chemoresistant, mitotic phenotype is a multi-stage process, but this hypothesis has not been systematically addressed as of yet.

However, the relationship of polyploidization/depolyploidization, cell cycle arrest, stemness, SASP and EMT is not fully understood. Thus, it is not clear, whether these processes occur in the same cell, and if so, whether they occur simultaneously or as a sequence of events. It is also not known whether giant cells are the central players in the formation of replicating, chemoresistant cells after application of DNA-damaging drugs or whether other cells play a major role.

In the present study, we have used the human ovarian carcinoma cell line SKOV3 [52], the most frequently used model for *in vitro* studies of ovarian cancer [53], to systematically address these questions. SKOV3 cells were originally described as being derived from an ovarian adenocarcinoma without specification of the histological subtype [52], but the subsequent analysis of xenotransplants in mice indicated a clear cell carcinoma origin [54]. This classification of SKOV3 cells is compatible with the presence of PIK3CA and ARID1A mutations, which are typical of human ovarian clear cell carcinoma, and the deletion rather than mutation of TP53 found in >97% of high grade serous adenocarcinomas [53, 55]. SKOV3 cells are moderately sensitive to CPT, but highly resistant cells can be selected for after drug exposure. Using this experimental system we found an ordered sequence of events that preceded the emergence of chemoresistance, which could essentially be recapitulated with TP53-mutated IGROV-1 cells, an ovarian cancer cell line most likely of low-grade serous adenocarcinoma origin [53, 56].

## RESULTS

### Proliferative CPT-resistant SKOV3 cells emerge after the transient occurrence of enlarged cells, polyploidy and accelerated senescence

**Figure 1:**
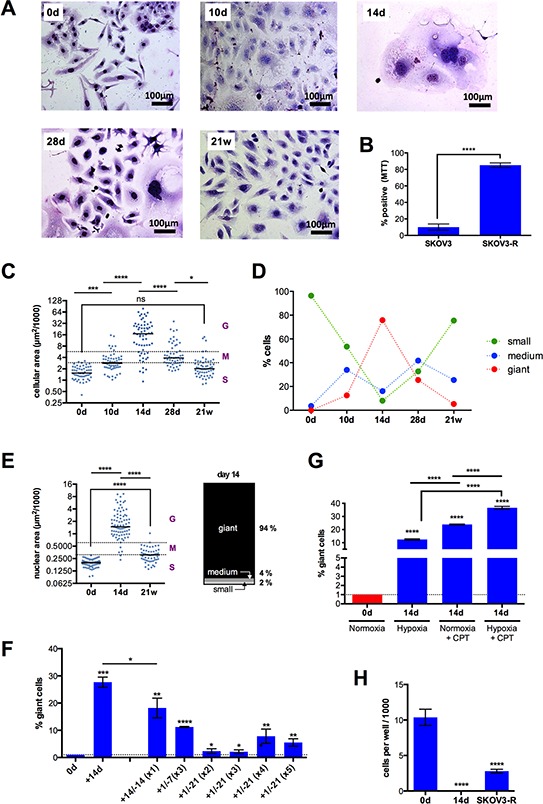
Morphology, size and growth properties of SKOV3 cells after CPT treatment **A.** Cells were treated with CPT for 10 days, 14 days, 28 days or 21 weeks, stained with Giemsa dye and evaluated by bright field microscopy. Representative photomicrographs are shown. **B.** Viability of SKOV3 and SKOV3-R cells after 14 days of CPT exposure (MTT assay). **C.** Size (area) of attached cells (*n* = 285) at the indicated times. Dotted lines define three populations distinguished by size; small (S), maximum the size of untreated SKOV3 cells; medium (M), maximum twice the size of small cells; giant (G), >4-times the size of small cells. The horizontal line shows the median. **D.** Time course of size distributions under CPT treatment. **E.** Nuclear area of cells (*n* = 204) at the indicated times. The stacked bar graph shows the distribution of giant, medium and small nuclei on day 14. **F.** Effect of cyclic CPT treatment. Cells were treated with CPT (days preceded by a “+” in the Figure) followed by a CPT-free recovery phase (days preceded by a “−”), and the respective cycle was repeated as indicated in parentheses (sample size: *n* = 3). **G.** Effect of hypoxia. Cells were treated with hypoxia, CPT or both for 14 days and the fraction of giant cells was determined by flow cytometry analysis. **H.** Anchorage-independent growth of untreated SKOV3 cells (0d) and cells treated with CPT for 14 days or 21 weeks. Cells were embedded in a collagen matrix after this treatment and observed under a phase-contrast microscope after another 12 days (sample size: *n* = 3). The cell count for “14d” was 0 in each experiment.

After an initial phase of cell death mainly resulting from mitotic catastrophe, as indicated by the interphase cells with multiple micronuclei, CPT-treated SKOV3 cells showed typical temporal alterations of cell morphology associated with profound changes in size, resulting in highly resistant cells after 21 weeks (Figure 1A, 1B; subsequently referred to as SKOV3-R cells). Median cell size of attached cells peaked at day 14 (16,000 μm^2^), and then progressively decreased to a size (2,000 μm^2^) only slightly larger than untreated cells (1.700 μm^2^). On day 14, the population consisted of a mixture of cell types which we defined as small (<3,000 μm^2^), medium (3,000–6,000 μm^2^) or giant cells (>6,000 μm^2^), with a distribution of 8%, 16% and 76%, respectively, the latter composed of mono- and polynucleated cells at a ration 2:1 (Figure 1C, 1D). The transient increase in cell size was also visible when detached cells were analyzed by flow cytometry (forward scatter; Figure S1). Another conspicuous feature of many of the larger cells appearing around day 14 was their flattened, senescent-like morphology. After day 14, the fraction of giant cells progressively decreased, while medium-sized cells first increased and then decreased and small cells continuously increased (Figure 1D). Since cell size not only depends on cell cycle phase and ploidy, we also determined the size of nuclei. As shown in Figure 1E, the changes in cell size were paralleled by similar changes in nuclear size (small : medium : giant cells = 2% : 4%: 94%;), pointing to a dynamic changes in ploidy during the observation period.

Giant cells are clearly the morphological hallmark of the cascade of events leading to chemoresistance and as such were subsequently used as a morphological marker for this process. Importantly, giant cells also emerged when cells were treated periodically with CPT, i.e., when short periods of treatment were followed by drug-free recovery phases, thus mimicking the clinical administration of chemotherapy. As illustrated in Figure 1F, substantial number of giant cells were observed after different time schedules, including 3 cycles of a 1-day treatment/7-day recovery regimen and 4 cycles of a 1-day treatment/21-day recovery schedule. These findings demonstrate that the continuous presence of CPT is not required to cause the formation of giant cells.

Giant SKOV3 cells have also been reported to be induced by hypoxia mimetics [43]. We were therefore interested to investigate whether hypoxia might have an impact on giant cell formation in response to CPT treatment. Figure 1G shows that hypoxia induced giant cell formation to a slightly lesser extent than CPT, thus confirming the results of the mimetics study quoted above. Intriguingly, the combination of both treatments showed a statistically highly significant cooperative effect.

Next, we compared the anchorage-independent growth potential in a collagen matrix of untreated SKOV3 cells, cells treated with CPT for 14 days and SKOV3-R cells. While untreated cells and SKOV3-R cells proliferated and formed colonies under these conditions, no surviving cells were detectable in cultures with SKOV3 cells treated with CPT for 14 days (Figure 1H). This indicates that (i) cell surviving a 14-day CPT treatment in “monolayer” cultures (as in Figure 1A) do not survive under anchorage-independent condition and (ii) SKOV3-R cells, on the other hand, have a similar capacity as the parental SKOV3 cells to survive in a collagen matrix, suggesting that this property is gained after day 14.

### Polyploidy and senescence in CPT-treated SKOV3 cells

**Figure 2:**
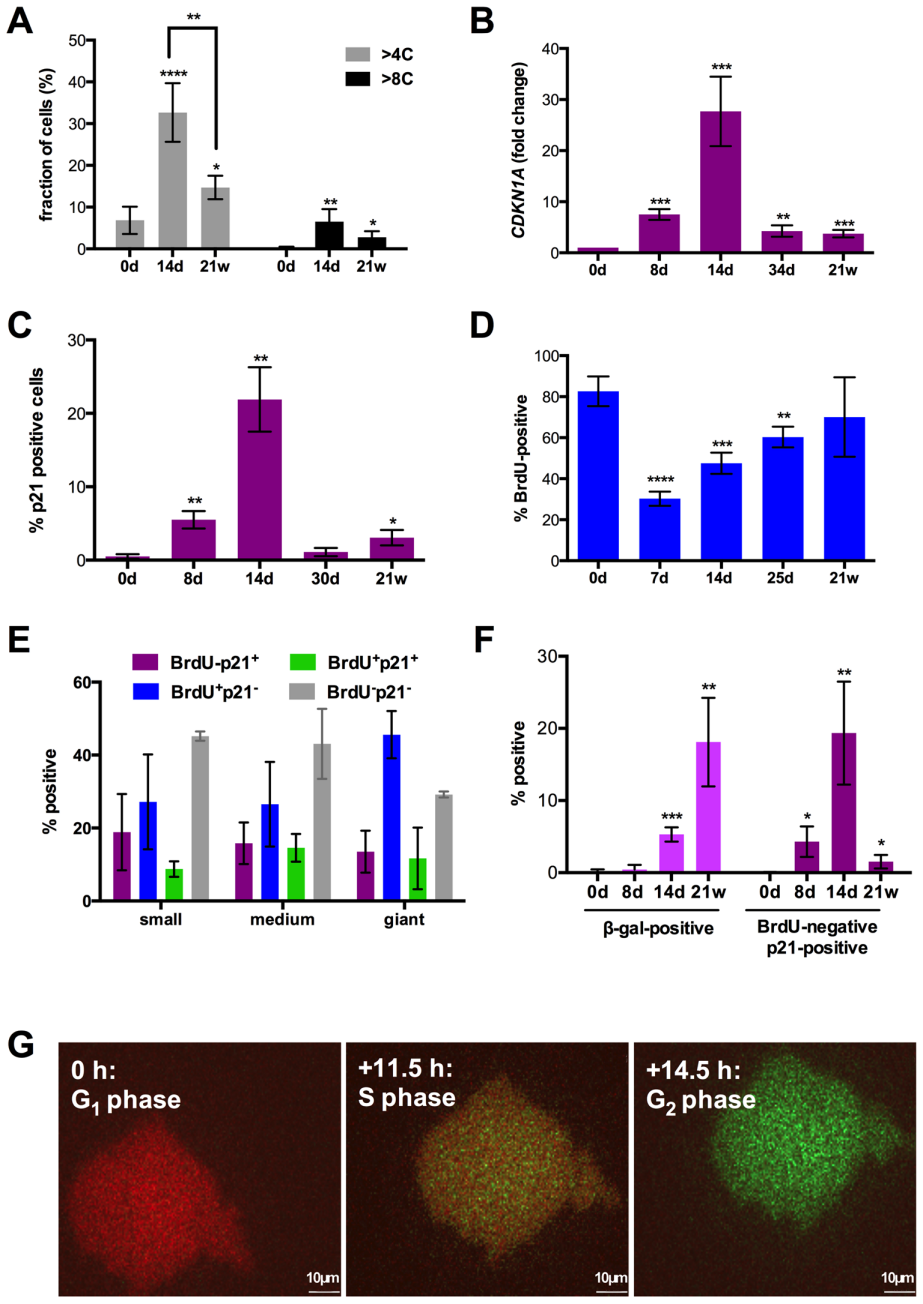
Cell cycle parameters after CPT treatment of SKOV3 cells **A.** Fraction of cells with a para-diploid (≤4C) or polyploid (>4C) DNA content at different times of CPT treatment determined by flow cytometry analysis after propidium iodide staining (sample size: *n* = 3). A corresponding histogram is shown in Figure S2. **B.** Quantitation of *CDKN1A* mRNA expression by RT-qPCR. **C, D.** Fractions of cells expressing p21 protein or incorporating BrdU visualized by immunofluorescence at the indicated times and microscopically quantified (sample size: *n* = 3). **E.** Fraction of small, medium and giant cells negative or positive for p21 expression and BrdU incorporation on day 14 (microscopic quantification; sample size: *n* = 3). **F.** Fraction of cells positive for β-galactosidase activity (X-Gal; left panel) or BrdU-negative and p21-positive (right panel; microscopic quantification; sample size: *n* = 3). **G.** SKOV3 cells on day 14 of CPT treatment infected with a baculovirus-derived vector expressing FUCCI cell cycle sensors. The Figure shows images of the same cell from a video obtained by life cell imaging at intervals of 11.5 (S) and 14.5 hours (G_2_) relative to the first image (G_1_).

In view of the profound changes in cell size under CPT treatment and previous reported induction of DNA endoreduplication and polyploidy by chemotherapy we analyzed the DNA content in relation to the size at different times of exposure to the drug. The flow cytometry analysis in Figures 2A and S2 show that CPT treatment clearly increased the fraction of SKOV3 cells with *a* > 4C or > 8C DNA content on day 14 (from 7% to 33% and from 0.3% to 6.5%, respectively), which decreased by approximately 50% after 21 weeks. An increased DNA content was also seen in smaller cells, indicating polyploidization after CPT treatment is not restricted to giant cells (Figure S2, middle panel).

CPT strongly induced expression of *CDKN1A* mRNA (Figure 2B) and its encoded protein p21 (Figure 2C) on day 8 with a peak on day 14. Thereafter, *CDKN1A* expression and the fraction of p21^+^ cells sharply declined, the latter from 22% on day 14 to 1% on day 30. The early increase in p21 expression on day 8 was paralleled by a decrease in 5-bromo-2′-deoxyuridine (BrdU) incorporation into the cellular DNA, which thereafter continuously increased until reaching approximately the level seen in untreated cells (Figure 2D). The increase in BrdU incorporation concomitantly with an augmented p21 expression on day 14 relative to day7/8 (Figure 2C, 2D) clearly suggests that the former is an indicator not only of S-phase progression but also of DNA repair.

Immunostaining of individual cells on day 14 clearly showed that p21^−^BrdU^+^, p21^+^BrdU^−^, double-positive and double-negative cells were found in cell populations of all three sizes at similar proportions (Figures 2E and S3). Previous studies have linked p21 to the butyrate-induced senescence of SKOV3 cells [57] and the ectopic expression of p21 induced a cell cycle arrest in SKOV3 cells [58]. It is therefore likely that the four cell populations identifies in Figure 2E represent cycling cells (p21^−^BrdU^+^), senescent cells (p21^+^BrdU^−^) and cell cycle arrested cells undergoing DNA repair (p21^+^BrdU^+^). The nature of p21^−^BrdU^−^ cells representing a major fraction in each size group is unclear. These cells are apparently cell cycle-arrested in the absence of high p21 levels, and may have entered a senescence-related state that mechanistically differs from the p21-dependent pathway. The data in Figure 1E (blue bars) also demonstrate that cycling cells are at readily detectable in each size group (27–47%).

On day 14, cells with increased lysosome β-galactosidase activity (β-gal^+^) were also detectable (Figure 2F), confirming the morphological observation of a substantial fraction of senescent cells at this time. In SKOV3-R cells, the fraction of p21^+^BrdU^−^ cells was decreased to 1.5% (Figure 2F). However, β-galactosidase activity was strongly increased in the mitotically active SKOV3-R4 cells, and there was no obvious morphological difference between β-galactosidase positive and negative cells. This suggests that the latter on is own in not an indicator of cellular senescence, as previously also suggested by others [59].

The senescence markers γH2AX and lack of Ki67 were not informative due to their induction by DNA damage [60] or cell cycle arrest [61], resulting in 100% positive cells in our experimental system. Another established marker of replicative senescence, p16ink4A, could not be analyzed due to a homozygous deletion of the *CDKN2A* gene in SKOV3 cells [62].

BrdU incorporation is not necessarily linked to cell cycle progression, but can also result from unscheduled DNA synthesis following DNA damage. We therefore infected SKOV3 cells with mammalian baculovirus vectors (BacMam) expressing the ubiquitination-based FUCCI cell cycle sensors, which label late S/G_2_ cells green (Geminin-GFP), G_1_ cells red (CTD1-REF) and early S-phase cells yellow [63]. Video S1 and Figure 2G shows the transition of the same giant cells from G_1_ through S into G_2_, indicating that giant SKOV3 cells emerging under CPT treatment are capable to progress normally through the cell cycle.

### Time-lapse life cell imaging of CPT-treated SKOV3 cells

**Figure 3:**
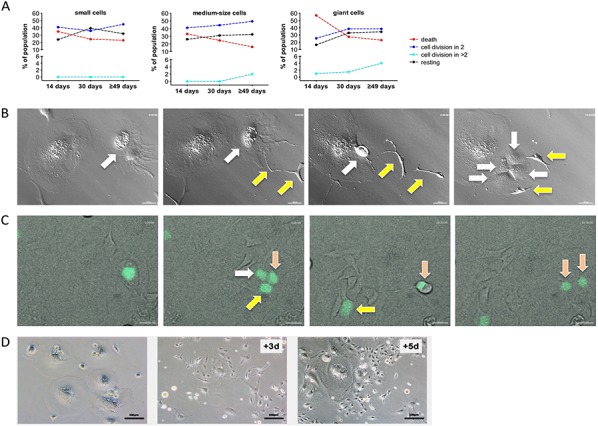
Time-lapse life cell imaging of CPT-treated SKOV3 cells **A.** Quantitation of cell fate on day 14, on day 30 and on day ≥49 of CPT treatment for small, medium and giant cells. Cells were observed for 70 hours by life cell imaging and grouped as indicated. Statistical analysis was performed by Chi-squared test. **B.** Images from Video S2 showing the budding of two cells (yellow arrows) from a giant cell (white arrow) followed by multi-daughter division of the giant cell into four daughter cells (white arrows). **C.** Images from Video S3 showing division of a giant cell into three daughter cells (white, yellow and pink arrows) followed by regular mitosis of one of the daughter cells (pink arrows). H2B-GFP expressing giant cells on day 14 of CPT treatment were enriched by filtering and plated on a layer of CPT-treated unlabeled feeder cells. **D.** Clonal out growth of small cells from giant cells. The images show an area initially occupied by giants cells (left-most image) and the same area 3 days and 5 days later.

To analyze the fate of individual cells with regard to the induction of cell death, division and depolyploidization we performed time-lapse microscopy of SKOV3 cells for 70 hours at three time points of CPT exposure (14 days, 30 days and 7–8 weeks). These studies identified three outcomes of treatment irrespective of cell size: death, division and no visible change (“resting”). Cell death was the main fate on giant cells on day 14 (60%), but clearly decreased on day 30 and thereafter, concomitantly with an increase in cell divisions exceeding that of cell death (Figure 3A). In small and medium size cells, differences in fate and time-related changes were less pronounced, suggesting an initial *status quo*, followed by an increase in cell number. Mitosis of mononuclear giant cells was usually followed by cytokinesis, and only rarely (<2%) resulted in the formation of multinuclear cells. However, ~17% of daughter cell underwent cell fusion immediately after cytokinesis, thereby creating new polyploid cells. Cell division of giant cells either resulted in two daughter cells or, less frequently, in ≥ 3 daughter cells (Figure 3B; Video S2) with a ratio of approximately 25:1 on day 14 and 10:1 after 49 days (Figure 3A). Furthermore, giant cells were able to generate daughter cells by budding (Figure 3B; Video S2). Importantly, cells generated by multi-daughter division were able to undergo subsequent bipolar divisions (Figure 3C; Video S3).

These observations suggest that initiation of CPT treatment is followed by an unstable, transient period that is characterized by the simultaneous occurrence of dividing, depolyploidizing, dying and resting cells. Between day 14 and 30 the balance is clearly shifted to survival and proliferation, consistent with the disappearance of p21^+^ cells (Figure 2C). The drop in p21^+^ cells is followed by a clear increase in depolyploidizing cells (Figure 3A), suggesting that p21^−^BrdU^+^ cells rather than senescent cells are undergoing depolyploidization.

Obviously, cells of all sizes can divide and thus have the potential to contribute to the subsequent development of chemoresistant, stably proliferating cells. However, in view of the low fraction of small cells present on day 14, a prominent role for the giant cells in the later emergence of SKOV3-R cells is very likely. This is supported by the microscopic observation in Figure 3D, showing rapid clonal growth of small cells in regions previously occupied by giant cells.

To assess the validity of the hypothesis that giant cells substantially contribute to the generation of SKOV3-R cells we modeled a mathematical simulation, which estimates the relative contribution of giant cells to the subsequent generation of small cells (Figure S4). The most crucial parameters of this simulation are the relative fractions of different size groups at different time points and the fractions of dividing and dying cells. These parameters can be extracted from the data in Figure 3A. Another important, albeit less decisive parameter is the average number of progeny per depolyploidizing cell. Evaluation of the video recordings showed that approximately 1/3 of these cells yielded 4 daughter cells and 2/3 gave rise to 3 daughter cells. Taking into account that additional cells were also generated by budding, we estimated the average total number of daughter cells generated per observation period (70 hours) as 3.7. A simulation performed on the basis of these parameters clearly shows the expected (see above) initial decline of giant cell numbers between day 14 and 29 and a more or less static situation for small cell numbers (Figure S4A). Furthermore, simulating the generation of small cells during the subsequent 30 days (Figure S4B) suggests that SKOV3-R cells originate to a considerable extent from giant cells, but also predicts a major contribution by small cells. However, it is likely that the latter are, at least in part, descendants of giant cells, pointing to an even greater contribution by the latter.

Taken together with the flow cytometric analyses of PI-stained cells described above, our observations reveal a ploidy conveyer, where the para-diploid cells undergo a G_2_ arrest, accumulate there, enter aberrant mitosis, by-pass mitotic catastrophe and then re-enter the cell cycle. This state of “cycling tetraploidy” has previously been proposed as a crucial step from diploidy to aneuploidy and from senescence to malignant transformation [40, 64, 65].

### Characterization of CPT-resistant SKOV3 cells by transcriptome analysis

**Figure 4:**
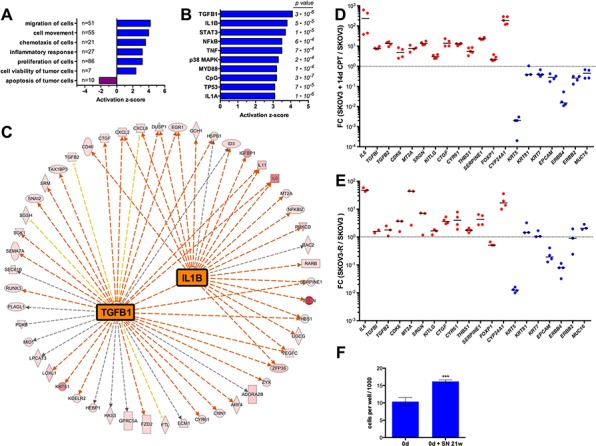
Transcriptome of SKOV3-R cells **A.** IPA Diseases and Functions Annotation of genes upregulated >10-fold in SKOV3-R cells versus normal SKOV3 cells. SKOV3-R cells were cultured in growth medium without CPT for 12 days prior to harvesting. The graph shows functionally different clusters with *p*-values < 0.001 and activation *z*-scores > 2 or < −2. Numbers to the right of the y-axis indicate the number of molecules in each category. **B.** IPA Upstream Regulator Analysis of genes upregulated in SKOV3-R cells. The graph shows the top regulators (by activation z-score) representing signaling molecules. Numbers on the right show the respective *p*-values. **C.** IPA network analysis of genes upregulated > 10-fold in SKOV3-R cells for the two top upstream regulators, TGFβ and IL-1β. Red arrows, induction consistent with prediction; yellow arrows, induction inconsistent with prediction; grey arrows, no prediction. The color intensity of the symbols indicates the strength of the observed induction (red highest). **D.** RT-qPCR validation of regulated genes in SKOV3 cells treated with CPT for 14 days versus untreated SKOV3 cells (sample size: *n* ≥ 3). FC, fold change. **E.** RT-qPCR analysis of gene expression in SKOV3-R versus SKOV3 cells (sample size: *n* ≥ 3). **F.** Anchorage-independent growth of untreated SKOV3 cells embedded in a collagen matrix in the presence or absence of conditioned medium from SKOV3-R cells (conditions as in Figure 1H).

As a first step to characterize the cells produced by long-term CPT treatment we compared the transcriptome of untreated SKOV3 and SKOV3-R cultured in the absence of CPT for 12 days. RNA-Seq analyses identified 314 genes upregulated in the latter (fold change > 10; Dataset S1). Diseases and functions annotation of these genes by Ingenuity Pathway Analysis (Figure 4A) showed a strong positive association (z-score > 2) with: (i) migration, cell movement and chemotaxis; (ii) inflammatory response; (iii) cell proliferation and (iv) cell viability, while an inverse association was seen for apoptosis (z-score < −2). Analysis of the known upstream regulators of these genes (signaling molecules and transcription factors) identified three main groups, i.e. TGFβ, pro-inflammatory mediators, such as IL-1B and TNF, and p53 (Figure 4B). As SKOV3 cells are *TP53*-deficient (homozygous deletion), the overlap of the latter group of genes with a subgroup of p53 target genes probably results from the induction of target genes by the DNA damage response through a p53-independent pathway. The genes of the IL1B and TNF networks partially overlap (Figure 4C) and indicate the upregulation of pro-inflammatory (e.g., *IL6, IL11, CXCL2*) and EMT markers (e.g., *SNAI2, TGFB2, RUNX3*). Furthermore, a dedifferentiated phenotype is suggested by the down-regulation of the cytokeratin genes *KRT5, KRT17* and *KRT81*, the epithelial markers *EPCAM* and *MUC16* (Dataset S1). These observations were confirmed by RT-qPCR for SKOV3-R cells cultured without drug in four independent experiments (Figure 4D). A very similar pattern was observed with RNA from SKOV3 cells treated with CPT for 2 weeks (Figure 4E), indicating that the observed changes represent relatively early events. Finally, the anchorage-independent growth of SKOV3 cells in a collagen matrix was stimulated by conditioned medium from SKOV3-R cells (Figure 4F), confirming the RNA-Seq-based prediction of a pro-tumorigenic secretome.

### Expression of stemness and pro-inflammatory markers in CPT-treated SKOV3 cells

**Figure 5:**
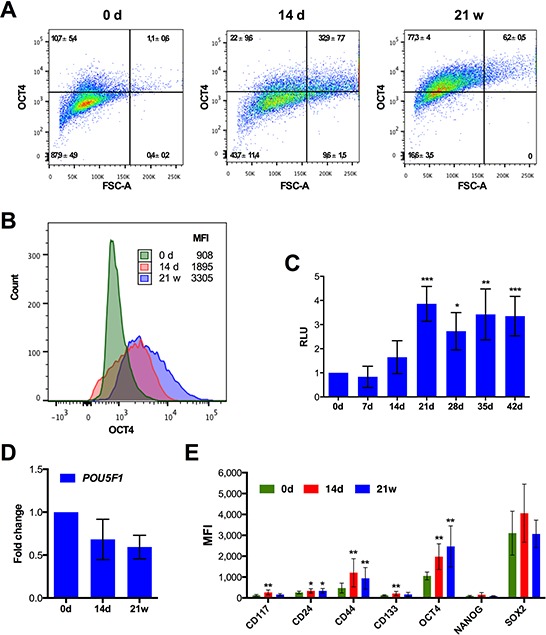
Expression of stemness markers after CPT treatment of SKOV3 cells **A.** Flow cytometry dot plot and **B.** histogram of nuclear OCT4 in untreated SKOV3 cells, SKOV3 cells treated for 14 days 21 weeks. The x-axis in panel A shows the forward scatter as an indication of cell size. A validation of the OCT4 antibody is shown in Figure S5. **C.** Quantitation of the transcriptional activity of OCT4 in SKOV3 cells harboring a stably integrated luciferase reporter construct driven by OCT4 binding sites after different times of CPT treatment. **D.** RT-qPCR analysis of *POU5F1* mRNA levels (sample size: *n* ≥ 3). **E.** Median fluorescence intensity (MFI) determined by flow cytometry for stem cell markers (CD24, CD44, CD117 and CD133 surface expression and nuclear OCT4, SOX2 and NANOG) after different times of CPT treatment (sample size: *n* ≥ 3).

Drug-induced senescence has been associated with stemness properties [40, 42, 66]. We therefore investigated the expression of stem cell markers in SKOV3 cells after different times of CPT treatment. Flow cytometry analysis showed a clear rise in OCT4^+^ cells on day 14 from 12% to 55%, which subsequently increased to 84% after 21 weeks (Figure 5A, 5B). Likewise, the transcriptional activity of OCT4 started to increase after 14 days of CPT treatment, reaching a plateau after 21 days, as determined with SKOV3 cells harboring an OCT4-responsive luciferase reporter plasmid (Figure 5C). Consistent with these findings, OCT4 has previously been reported to upregulated during reversible polyploidy following DNA damage of cells lacking wild-type p53 [41, 67, 68]. Intriguingly, the increase in OCT4 protein level and activity was not paralleled by an increased level of *POU5F1* (OCT4) mRNA (Figure 5D), pointing to a regulatory mechanism operating at the level of translation.

We also determined expression of several other stem cell markers (Figure 5E). CD44, CD117 and CD133 also showed significantly increased median fluorescence intensities (MFI) on day 14 of CPT treatment. CD44 levels remained elevated after 21 weeks, whereas expression of the two other markers dropped. No significant changes in NANOG and SOX2 expression were detectable with SOX2 being expressed at high level already prior to treatment (Figure 5E).

**Figure 6:**
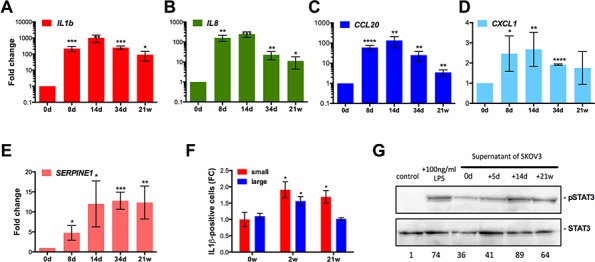
Induction of a pro-inflammatory secretory phenotype after CPT treatment of SKOV3 cells **A–E.** RT-qPCR expression analysis of the indicated pro-inflammatory marker genes at the indicated times of CPT treatment (sample size: *n* ≥ 3). FC = 1 (day 0) corresponds to the following Ct values: *IL1B:* 31.5; *IL8:* 32; *CCL20:* 32; *CXCL1*: 22; *SERPINE1:* 26. **F.** Flow cytometry analysis of intracellular IL-1β expression after 0, 2 or 21 weeks of CPT treatment (sample size: *n* = 3). Values represent the fold change (FC) in the number of positive cells relative to small cells on day 0 (normalized to 1). Cells gated for small cells (forward and sideward scatter as untreated SKOV3 cells) and larger cells (forward and sideward scatter larger than for SKOV3 cells). **G.** Immunoblot analysis of phosphorylated STAT3 in untreated human macrophage cells (negative control), after LPS treatment (positive control), or after an exposure to supernatant from SKOV3 cells treated with CPT for 0, 5 days, 14 days or 21 weeks.

The pro-inflammatory phenotype of SKOV3-R cells and the emergence of senescent cells following drug treatment pointed to the induction of a SASP. We therefore analyzed the expression of several SASP marker genes and proteins after treatment of SKOV3 cells with CPT. The *IL1B, IL8* and *CCL20* genes are strongly induced on day 14 (up to 1000-fold; Figure 6A–6C). Expression declined thereafter, but was still clearly elevated in SKOV3-R cells. Two other SASP marker genes, *CXCL1* and *SERPINE1*, were induced moderately (2- to 10-fold), and remained at these levels throughout the observation period (Figure 6D–6E). Consistent with the RNA expression data, flow cytometry showed an increase in the fraction of intracellular IL-1β^+^ cells on day 14 and a decline thereafter (Figure 6F). Gating of cells according to forward and sideward scatters showed that small and large cells only slightly differed in the levels of intracellular IL-1β, indicating that the expression of pro-inflammatory marker genes in SKOV3-R cells do not result from a small subpopulation of giant cells. The induction of a pro-inflammatory secretory phenotype was also functionally confirmed by demonstrating an increased phosphorylated STAT3 in a human macrophage cell line (differentiated THP-1 cells) by supernatant from SKOV3 cells treated with CPT for 14 days or 21 weeks (Figure 6G).

### Progressive EMT of CPT-treated SKOV3 cells

**Figure 7:**
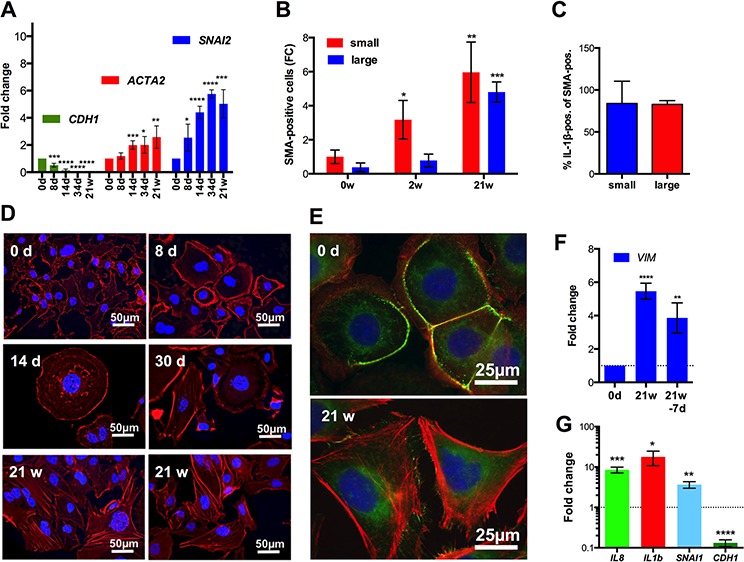
Induction of EMT after CPT treatment of SKOV3 cells **A.** RT-qPCR expression analysis of the indicated EMT marker genes after the indicated times of CPT treatment (sample size: *n* ≥ 3). **B.** Flow cytometry analysis of intracellular SMA after 0, 2 or 21 weeks of CPT treatment (sample size: *n* = 3). Values represent the fold change (FC) in the number of positive cells relative to small cells on day 0 (normalized to 1). Cells gated for small cells (forward and sideward scatter as untreated SKOV3 cells) and larger cells (forward and sideward scatter larger than for SKOV3 cells). **C.** Flow cytometry analysis of IL-1β expression in SMA-expressing SKOV3-R cells gated as in panel B. **D.** Labeling of actin filaments with California Red-conjugated phalloidin (red) after different periods of CPT exposure. **E.** Additional staining of the tight junction (zonula occludens) protein constituent ZO-1 by indirect immunofluorescence (green) in untreated SKOV3 and SKOV3-R cells. Nuclei were stained with DAPI in panels D and E. **F.** RT-qPCR analysis of *VIM* expression in SKOV3-R cells (21w) and the same cells grown in the absence of CPT for 7 days. **G.** Pro-inflammatory and EMT marker gene expression after cyclic CPT treatment (1 day CPT, 21 days recovery, 4 cycles) compared to untreated cells (sample size: *n* = 3).

The induction of a signaling network characteristic of TGFβ, the induction of *SNAI2* and the decreased expression of epithelial marker genes in SKOV3-R cells strongly suggested a phenotypic conversion of CPT-treated cells by EMT. RT-qPCR expression analysis of several EMT marker genes confirmed this conclusion (Figure 7A). While *CDH1* (E-cadherin) showed a strong progressive decrease beginning between day 8 and 14, *ACTA2* (smooth muscle α-actin) and *SNAI2* (SLUG) mRNA expression strongly and progressively increased until week 21. Analysis of intracellular SMA by flow cytometry showed a progressive rise until 21 weeks in both small and large cells (Figure 7B) and thus is consistent with the RNA data. Furthermore, as demonstrated by the flow cytometry analysis in Figure 7C, most cells expressing SMA above the detection limit (isotype control) co-expressed IL-1β in SKOV3-R cells (Figure 7C). Conversely, a substantial fraction of IL-1β expressing cells co-expressed SMA, although the overlap was lower in this case, presumably due to differences in the detection limits of the antibodies used. These observations indicate that induction of a pro-inflammatory secretory phenotype and EMT occurred simultaneously in a large fraction of the SKOV3-R cells.

The induction of EMT was also confirmed by staining actin filaments with fluorescently labeled phalloidin, which showed the formation of actin stress fibers around day 30 and increasing thereafter (Figure 7D). Consistent with this observation, staining of the zonula occludens protein constituent ZO-1 by indirect immunofluorescence revealed a loss of tight junctions in SKOV3-R cells (Figure 7E).

Increased cell motility and migration have been associated with EMT in several model systems [69]. We therefore analyzed these parameters for SKOV3-R versus the parental SKOV3 cells. Life cell video microscopy revealed a strikingly increase in the motility of SKOV3-R cells, as shown in Video S4 (SKOV3) and Video S5 (SKOV3-R). Likewise, real-time electrical impedance measurements showed a clearly accelerated directional migration of SKOV3-R cells towards a chemoattractant (FCS; Figure S6). These findings strongly suggest that EMT is linked to increased cell motility and migration in our experimental model.

Importantly, RT-qPCR analysis of SKOV3-R cells grown in the absence of CPT for 7 days only showed a marginal effect of the drug withdrawal on the expression of the EMT maker gene *VIM* (vimentin; Figure 7F). Furthermore, SKOV3 cells after cyclic CPT treatment (4 cycles of 1 day CPT and 21 days recovery) also resulted in an up-regulation of *IL1B, IL8* and *SNAI1* and down-regulation of *CDH1* (Figure 7G). These findings indicate that induction of a pro-inflammatory secretory phenotype and EMT are not merely a consequence of chronic drug exposure.

### IGROV-1 ovarian carcinoma cells follow a similar multi-stage process to acquire chemoresistance

**Figure 8:**
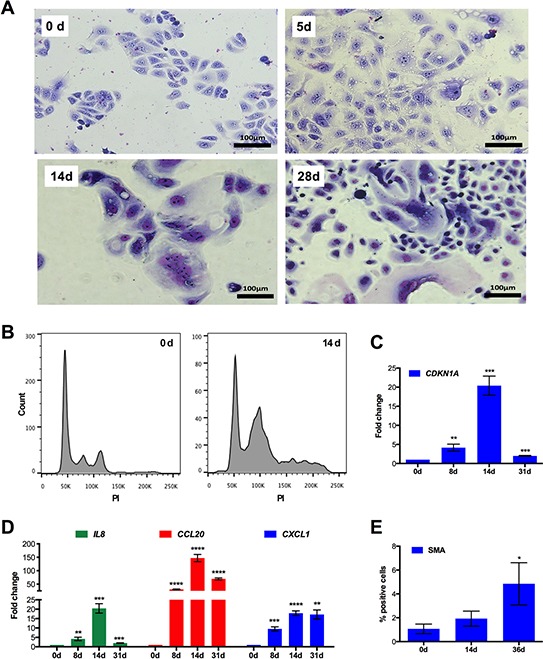
Multi-stage process of chemoresistance acquisition by IGROV-1 cells **A.** Morphology of cells that were treated with CPT for 5 days, 14 days and 28 days. Representative photomicrographs of cells stained with Giemsa dye are shown. **B.** DNA content of individual cells after CPT treatment for 14 days. Cells were stained with propidium iodide and analyzed by flow cytometry (sample size: *n* = 3). **C, D.** RT-qPCR analysis of *CDKN1A* (panel C) and pro-inflammatory marker genes (panel D) after different times of CPT treatment (sample size: *n* = 3). **E.** Expression of SMA protein determined by flow cytometry after the indicated periods of CPT exposure (sample size: *n* = 3).

Finally, we were able to demonstrate that a similar CPT-induced multi-stage process also occurs in another ovarian cancer cell line, IGROV-1. As depicted In Figure 8A, IGROV-1 cultures also contained substantial numbers of giant (Figure 8A) and polyploid cells (Figure 8B) on day 14 of drug treatment. Moreover, as observed with SKOV3 cells, *CDKN1A* and the pro-inflammatory marker genes *IL8, CCL20* and *CXCL1* were induced with a peak around day 14 (Figure 8C and 8D), which was followed by the up regulation of SMA (Figure 8E).

## DISCUSSION

Besides different forms of programmed cell death multiple other mechanisms have been identified to mediate the cytotoxic effect of chemotherapy or radiation on cancer cells in solid tumors, including mitotic catastrophe and permanent cell cycle arrest resembling cellular senescence [70]. In contrast to replicative senescence responsible for the limited lifespan of normal cells, drug-induced senescence occurs in replication-competent cells and induces a prolonged, but not necessarily permanent cell cycle block. This response, also referred to as accelerated senescence, is associated with the formation of polyploid giant cells that are endowed with the potential to form para-diploid progeny. Several studies have shown the upregulation of stemness markers in giant cells, pointing to cancer-initiating properties of these cells or their descendants. Giant cells thus might provide a mechanism by which cancer cells can escape therapy-induced cell death and contribute to relapse of the disease by converting to proliferating cells after depolyploidization.

**Figure 9:**
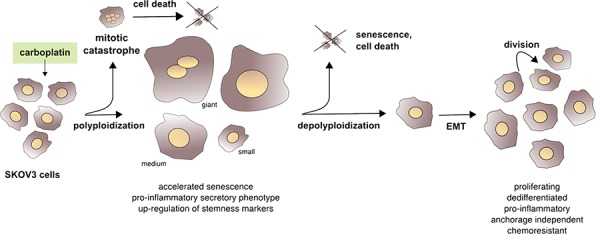
Summary scheme After the acute induction of cell death in most of the cells, CPT triggers a sequence of partially reversible events, including polyploidization and accelerated senescence. This is accompanied by a more stable expression of stemness markers and the acquisition of a pro-inflammatory secretory phenotype, followed by EMT and the emergence of proliferative, anchorage-independent, dedifferentiated and pro-inflammatory chemoresistant cells.

DNA-damaging chemotherapeutic drugs can also trigger other processes linked to senescence, such as SASP and EMT. However, prior to the present study it was unclear how these events are temporally and spatially linked to each other and to the development of CPT resistance, and to what extent giant and non-giant cells contribute to this process. We have addressed these question using ovarian cancer cells as a model. Our data (summarized in Figure S7) show that after the acute induction of cell death by mitotic catastrophe in the majority of the cell population, cells of different sizes and ploidy, including giant and para-diploid cells, survive. These cells show cell cycle perturbations resembling accelerated senescence, which is accompanied by the persistent expression of the stemness marker OCT4 and the acquisition of a pro-inflammatory status. At the same time cells begin to undergo EMT, which progressively increases thereafter and is maintained in the cells finally emerging from this multi-stage process. CPT thus triggers a sequence of partially reversible events, including the transient formation of polyploid giant cells. This integrated picture of events leading to the emergence of proliferative chemoresistant cells is summarized in the scheme depicted in Figure 9. Several key issues in the context with this model are discussed in detail below.

A key question is the question whether resistant cells result from conversion rather than selection of preexisting cells. At least two observations argue in favor of the first possibility. In day 14 cultures the number of small cells is very low and the rates of cell division and death are similar (Figure 3A), making an expansion of these cells in the following days unlikely. Giant cells are present at much larger numbers at the same time, but their death rate exceeds the rate of division. This results in a scenario on day 28 where small and giant cells are present at similar numbers, as suggested by the microscopic evaluation in Figure 1C and 1D. On day 30 and thereafter, the death rate of giant cells is strongly decreased and falls below the rate of division, while the fate of small cells remains largely unchanged (Figure 3A). Between day 14 and 30, giant cells already undergo multi-daughter divisions into more than two daughter cells (Figure 3A), which presumably results in their depolyploidization. Thus, small cells present on day 30 but generated from giant cells before day 30 will contribute to the expansion of the pool of small cells. This is consistent with the simulation in Figure S4B, which suggests that giant cells present on day 30 contribute to the subsequent generation of small cells, making it very unlikely that the selection of preexisting small cells in the original SKOV3 population are the origin of the small SKOV3-R cells eventually emerging at later stages. The possibility that preexisting giant cells are selected is similarly unlikely in view of their dramatic increase between day 10 and 14 (Figure 1C), in spite of their low rate of division and a high rate of cell death (Figure 3A), which strongly suggests that these cells arise from smaller cells by endoreduplication and/or cell fusion. Another finding arguing against the selection hypothesis is the fact that cells surviving a 14-day drug treatment are unable to seed in a collagen matrix, indicating that cells with the properties of SKOV3-R are not present at this stage.

While these observations assign an essential role to giant cells in the formation of SKOV3-R cells, a significant contribution by medium-size cells is less likely. This is suggested by the absence of any detectable depolyploidizing events in day 14 and day 30 cultures, indicating that multi-daughter divisions occur less frequently in these cells than in giant cells. At later stages, divisions of medium-size cells into more than two daughter cells are observed (Figure 3A). Although these still occur at a lower frequency than in giant cells, it is likely that medium-size cells contribute to the emergence of SKOV3-R cells at later stages. Taken together, these observations point a key role of polyploid cells, in particular giant cells, in the generation of proliferating, chemoresistant SKOV3 cells.

Another critical issue concerns the role of senescence. Previous studies have provided evidence that DNA damaging chemotherapeutic drugs induce polyploidy, which is reversible in a small subpopulation of these cells, resulting in the emergence of resistant, clonogenic malignant cells (reviewed in [40]). The same studies also suggested that polyploidization and depolyploidization are linked to the induction and reversion of accelerated senescence. Our own data suggest that senescence and polyploidy are not necessarily linked in a large fraction of the cells. Thus, p21^+^BrdU^−^ cells occur to a similar extent in large, medium-size and giant cells (Figure 2E), indicating that senescence occurs irrespective of ploidy, including small para-diploid cells. Furthermore, we identified a large fraction of giant cells that undergo regular cell division into two daughter cells (Figure 3A), presumably representing the p21^−^BrdU^+^ cells in Figure 2E. In addition, only about 5% of the cells on day 14 are positive for β-galactosidase (Figure 2F), although giant cells prevail at this time. These observations suggest that: (i) the induction of polyploidy in SKOV3 cells by CPT is not strictly dependent on the induction of senescence; and (ii) small, para-diploid cells can originate from replication-competent polyploid giant cells. This certainly does not exclude the possibility that rejuvenating senescent giant cells also generate para-diploid, cycling cells, as previously described in other experimental systems [40], perhaps through a replicating giant cell as an intermediate. This matter is very complex and needs more research to be able to draw definitive conclusions as to the role of senescent cells in reversible polyploidization.

Our data also show that the combination of CPT treatment with hypoxia produces a cooperative effect with respect to the generation of giant cells. A recent publication described the induction of giant SKOV3 by hypoxia mimetics [43], but the observed cooperative effect with a chemotherapeutic agent has not been reported to date. Several previous studies have addressed the link between hypoxia and senescence, which is also relevant for the present study. While an inhibitory effect of hypoxia on senescence has been observed with most experimental models, there are also opposing findings with other systems, making this a highly controversial issue [71–78]. The situation is further complicated by the fact that the linkage of senescence and hypoxia is dependent on p53, at least in some experimental systems [77], making it difficult to extrapolate the published findings to SKOV3 cells lacking *TP53* genes. However, if hypoxia indeed suppresses senescence of SKOV3 cells as in the majority of experimental systems, this would support the conclusion that senescence is not a prerequisite for giant cell formation.

Our data also show the upregulation of pro-inflammatory markers on day 14 of CPT treatment (Figure 6). However, at least in the case of IL-1β this occurs in the majority of cells (Figure 6F) and therefore does not appear to be restricted to senescent cells. It thus seems appropriate to refer to this status as pro-inflammatory secretory phenotype rather than SASP. Intriguingly, these markers remain upregulated in SKOV3-R cells, both with and without ongoing CPT treatment (Figures 6 and 8D), indicating that the pro-inflammatory secretory status triggered around day 14 is maintained in the cycling resistant cells emerging at later stages. It is likely that the secretion of inflammatory cytokines has no direct impact on the multi-stage process described, but rather contributes to drug resistance and an increased malignancy of the resulting cells. A striking feature of the finally emerging SKOV3-R cells is their high β-galactosidase activity (Figure 2F), a hallmark of senescent cells in other systems. It thus appears that SKOV3-R combine features of senescence, including a pro-inflammatory phenotype and β-galactosidase activity, with low p21 expression and a high mitotic activity.

EMT is another process previously linked to chemoresistance, but its relationship to other events has not been systematically addressed. In the model system investigated in the present study, EMT temporally differs from all processed analyzed and discussed above, since it progressively increases after day 14. EMT thus appears to parallel the emergence of SKOV3-R cells. This is suggested by an up-regulation of EMT marker genes concomitantly with the repression of the E-cadherin encoding gene *CDH1* in SKOV3-R cells (Figure 7A), a dramatic increase of SMA-positive cells (Figure 7B), the occurrence of actin stress fibers (Figure 7D) and the loss of tight junctions (Figure 7F). The data in Figure 4 also show that multiple epithelial marker genes are strongly repressed in SKOV3-R cells, indicating that these cells are dedifferentiated, which is consistent with the occurrence of EMT. Flow cytometry of SMA expression also showed that EMT was not restricted to a small subpopulation of the heterogeneous SKOV3-R cell population, but occurred in the vast majority of these cells (Figure 7B). Furthermore, increased SMA and IL-1β expression were found in the same cells (Figure 7C), indicating that EMT occurred in cells with a pro-inflammatory secretory phenotype.

Finally, our data show that four stemness markers were up-regulated during the observed multi-stage process (Figure 5E). OCT-4, CD44, CD117 and CD133 showed a clear increase on day 14. While expression of the latter two was elevated transiently and coincided with the maximal occurrence of polyploid and giant cells, OCT-4 and CD44 remained elevated at later stages, including SKOV3-R cells. It is thus possible that CD117 and CD133 play a role in polyploidization, which would be in line with the previously proposed link of stemness to the transient formation of polyploid giant cells [40]. In contrast to OCT-4, expression of the functionally related transcription factors NANOG and SOX2 did not change, consistent with a previous report on etoposide-treated PA-1 cells [79]. This suggests that OCT-4 fulfills a function unrelated to self-renewal in drug-treated cancer cells. In the PA-1 model alluded to above, OCT-4 was associated with the up-regulation of p21 expression. It appears that this does not apply to our experimental system, since *CDKN1A* and p21 expression (Figure 2B, 2C) is inversely correlated with OCT-4 levels (Figure 5). Nevertheless, OCT-4 appears to be of particular interest, since its expression and activity are elevated in SKOV3-R cells, raising the possibility that it plays a role in conferring CPT resistance, as reported for drug-treated hepatoma cells [80]. We therefore attempted to analyze the function of OCT-4 in CPT-treated SKOV3 cells by silencing of *POU5F1* mRNA, but were unable to obtain SKOV3 cells with substantially reduced level of OCT-4 protein after lentiviral shRNA transduction (not shown), suggesting that OCT-4 plays an essential role in these cells.

Collectively, these findings led to the model in Figure 9, depicting the time course of events eventually leading to cycling, chemoresistant SKOV3 cells that are capable of growing in an anchorage-independent manner and display a dedifferentiated and pro-inflammatory secretory phenotype. Importantly, key events of these hallmarks of malignancy were also seen in IGROV-1 cells and in SKOV3 cells exposed to cyclic CPT treatment, including a regimen of 1 day exposure and 21 days recovery, mimicking the clinical application of chemotherapy. It is thus possible that the multi-stage process described here also occurs in a clinical setting and contributes to the emergence of chemoresistant cells and perhaps the generation of dormant cells that escaped drug-induced death by polyploidization and subsequently rejuvenate to cause relapse of the disease long after the initial therapy.

## MATERIALS AND METHODS

### Cell culture

SKOV3 cells were obtained from the ATCC. H2B-GFP-SKOV3 cells were generated by stable transfection of SKOV3 cells with pBABE-H2GFP (Addgene). IGROV-1 cells were a kind gift of T. Hagemann (London). SKOV3 cells were cultured in McCoy's 5a (Life Technologies), complemented with 10% fetal calf serum (PAA, Sigma), 100 U/ml penicillin, and 100 mg/ml streptomycin (Sigma). IGROV-1 cells were cultured in RPMI 1640 (Life Technologies), complemented with 10% fetal calf serum (PAA, Sigma), 100 U/ml penicillin, 100 mg/ml streptomycin (Sigma) and 2 g/l NaHCO_3_. THP1 cells (ATCC, TIB-202) were cultured in RPMI 1640 (Life Technologies), complemented with 10% fetal calf serum (PAA, Sigma) and 0,05 mM β-mercaptoethanol. Cells were maintained in a humidified incubator at 37°C and 5% CO_2_. To monitor cell cycle progression 5 × 10^4^ cells were transduced two hours after plating with FUCCI Cell Cycle Sensor [63] expressed by the mammalian baculovirus vector BacMam 2.0 (Premo FUCCI; Life Technologies) at an infection multiplicity of 50 particles per cell and subsequently cultured for 24 h. For some experiments giant SKOV3 cells were enriched by filtering though 30 μm filters (Miltenyi Biotech).

### Cell viability assay

Equal cell numbers were seeded into 96-well plates and treated as indicated. 3-[4,5-dimethylthiazol-2-yl]-2,5-diphenyl tetrazolium bromide (MTT; Sigma) was added at a final concentration of 0.5 mg/ml and the plates were incubated for 4 hours at 37°C. MTT formazan crystals were dissolved with 5% sodium dodecyl sulphate + 0.005 M HCl for another 4 hours at 37°C. Color development was measured at 570 nm.

### Time-lapse life cell video microscopy

Live cell imaging movies were recorded for 70 h with a resolution of 1388 × 1040 pixels, one picture per hour and on average 14 regions / time period using a Axiovert microscope equipped with a CO_2_ incubator and a 10x DIC objective (Zeiss). H2B-labeled cells were tracked with an Axio Observer spinning disk microscope with 10× objective (Zeiss) at a resolution of 512 × 512 pixels, one picture per 30 minutes and a recording time of 67 hours. Movies of cells transfected with FUCCI cell cycle sensors were recorded for 64 hours by spinning disc microscopy with a 25× objective, one picture / 15 minutes and a 512 × 512 pixel resolution.

### Anchorage-independent growth of spheres in a collagen matrix

A mixture of 2 mg/ml collagen type I (rat tail, Life) and 8 mM NaOH in medium was prepared on ice and adjusted to pH 6.5–7. Fifty microliters of the neutralized solution were pipetted into a 96 well plate and allowed to polymerize for 5 minutes. Then, 5,000 cells per well were mixed with 75 μl of the neutralized collagen solution and added to the coated wells. After 1 h at 37°C, 100 μl medium were added and changed once a week. The assay was evaluated under a phase-contrast microscope after two weeks.

### CPT treatment and hypoxia

CPT (Santa Cruz) was used at a final concentration of 6 μM. For hypoxia experiments cells were kept at 1% O_2_ for 2 weeks.

### Flow cytometry

After harvesting by trypsinization cells were washed with phosphate buffered saline (PBS). Intracellular staining was performed after permeabilization for 60 min at 4°C using Foxp3/ Transcription Factor Staining Buffer Set (eBioscience). Staining was performed at 4°C 30 min for PerCPT-Vio700-labeled CD24 (Miltenyi Biotec, 130–097-914), VioBlue-labeled anti-CD44 (Miltenyi Biotec, 130–099-268), APC-labeled anti-CD133 (Miltenyi Biotec, 130–090-826), PE-labeled anti-CD117 (BD Biosciences, 555714), PE-labeled anti-NANOG (BD Biosciences, 560873), anti-SOX2 (Millipore, AB5603), anti-OCT4 (Santa Cruz, sc9081), PE-labeled IL1β (eBioscience, 12–7018-41), FITC-labeled anti-SMA (Sigma, F3777) and FITC-labeled anti-rabbit IgG (eBioscience, 11–4839) and PE-labeled anti-rabbit IgG (eBioscience, 12–4739) were used as secondary antibodies, if required. Isotype control antibodies were obtained from BD Biosciences, Biozol and eBioscience. Cells were analyzed using a FACS Canto flow cytometer and FACS Diva or FlowJo software (BD Biosciences). Results were calculated as percentage of live cells and mean fluorescence intensity (MFI). For propidium iodide (PI) staining cells were harvested and resuspended in 300 μl PBS. 700 μl ice-cold 100% ethanol was added slowly under gentle mixing. After incubation of at least 1 h at 4°C cells were pelleted and incubated for 30 min with 10 μg/ml RNase A and 20 μg/ml PI (Sigma) at room temperature in the dark. Cells were analyzed by flow cytometry as above.

### BrdU incorporation

Cells were labeled with 4 μg/ml BrdU for approximately 18 hours. After washing with PBS cells were fixed with 75% ethanol (−20°C) for 15 min. Subsequently, cells were treated with 1 M HCl for 1 h at 37°C. They were then treated twice with sodium borate for 5 min. After incubation with 10% BSA in PBS for 20 minutes at room temperature they were incubated with a mouse anti-BrdU antibody (1:20; BD Biosciences) at room temperature for 1 h. PBS washes were performed between each step. Cells were incubated with anti-mouse conjugated to Alexa 488 (1:400, Invitrogen) for 1 h. The nuclei were counterstained with DAPI. For combination with p21 staining, cells were incubated after sodium borate treatment with mouse anti-BrdU antibody (as above) plus rabbit anti-p21 antibody (1:100; Cell Signaling, #2947) at room temperature for 1 h, followed by Alexa 488-conjugated anti-mouse IgG (1:400, Invitrogen) and Alexa 594-conjugated anti-rabbit IgG (1:400, Invitrogen) for 1 h.

### Immunostaining

Cells were fixed with formaldehyde (4%), permeabilized by 0,1% Triton X-100 (5 min) and stained by using a FITC labeled-ZO-1 antibody (Invitrogen, 339188) and Phalloidin (California Red Conjugate, Biomol). Cells were counterstained with Vecatshield including DAPI (Biozol). Slides were evaluated with a Leica RMB 3 microscope equipped with fluorescence optics.

### β-galactosidase and giemsa staining

Cells were fixed with formaldehyde (4%) for 5 min at room temperature and washed with PBS. Staining solution containing 0,1% X-gal, 5 mM potassium ferrocyanide, 5 mM potassium ferricyanide, 150 mM sodium chloride, 2 mM magnesium chloride in 40 mM citric acid/sodium phosphate solution (pH 6) was added to the cells and an incubation period of 24 h at 37°C followed. To terminate the reaction staining solution was replaced with distilled water. Blue-stained SA-β-gal positive cells were counted as a percentage of the total cell number. For Giemsa staining cells were fixed with methanol at room temperature for 5 min, incubated with 10% Giemsa staining solution for 10 min and washed several times with distilled water.

### Luciferase assay

SKOV3 cells were transduced with a lentivector-based reporter construct that is responsive to OCT4 activity via OCT4 response elements and expresses destabilized green fluorescent protein and firefly luciferase from the mCMV promoter (pGF1-mCMV; System Biosciences). For determination of luciferase activity, cells were lysed with a lysis buffer containing 77 mM K_2_HPO_4_, 23 mM KH_2_PO_4_ and 0,2% Triton X-1000. Measurement was done according to the instructions of Beetle-Juice Big-Kit (PJK). Light units of firefly luciferase were normalized to protein concentration, using a commercial protein assay kit (BioRad).

### Analysis of phospo-STAT3 induction

THP1 cells were differentiated to macrophages by an overnight treatment with 5 ng/ml of TPA (Sigma). Two days later cells were treated with 100 ng/ml lipopolysaccharide (Sigma) as a positive control, or with supernatant from untreated SKOV3 cells or SKOV3 cells exposed to carboplatin. After 24 h cells were lysed in 50 mM Tris pH 6.8, 2% SDS, 6% glycerin and 1% β-mercaptoethanol. Cell lysates were subjected to SDS-PAGE on 12% gels and immunoblotting was performed using the following antibodies: anti-STAT3 (124H6, Cell Signaling, #9139), anti-phospho-STAT3 (Tyr-705, Cell Signaling, #9145), horse reddish peroxidase-linked anti-rabbit IgG (Cell Signaling, #7074) and horse reddish peroxidase-linked anti-mouse IgG (eBioscience, 18–8817-33).

### RT-qPCR

cDNA was synthesized from 0,1–1 μg of RNA using the iScript kit (Biorad, Germany). qPCR was performed in a Mx3000P Real-Time PCR system (Stratagene, La Jolla, CA) for 40 cycles at an annealing temperature of 60°C. PCR reactions were carried out using the Absolute QPCR SYBR Green Mix (Thermo Fisher) and a primer concentration of 0,2 μM following the manufacturer's instructions. L27 was used as normalizer. PCR primer sequences are listed in Table S1.

### RNA sequencing (RNA-Seq) and data analysis

RNA extraction, sample preparation for RNA-Seq and sequencing on an Illumina HiSeq 1500 were performed and have been published in detail elsewhere [81]. RNA-Seq data were aligned to Ensembl v74 using STAR (version STAR_2.3.1z13_r470) [82]. Determination of gene read counts, normalization and calculation of FPKM (fragments per kb per million) were carried out as described [81]. Genes were considered for further analysis if they had a minimum FPKM of 1 in any condition and at least 50 raw reads. Data were analyzed using Ingenuity Pathway Analysis (IPA; Qiagen Redwood City, CA, USA). The functions Upstream Regulators and Diseases and Bio Functions were applied using the default settings. RNA-Seq datasets were deposited at EBI ArrayExpress under accession number E-MTAB-3770.

### Statistical analyses

All experiments were carried out as independent biological replicates (*n* ≥3); the exact sample sizes are indicated in the Figure legends. Bar plots show the standard deviation (error bars) and the statistical significance determined by Student's *t*-test (unpaired, two-sided, equal variance) using GraphPad Prism 6.0 as follows: *p < 0.05; **p < 0.01; ***p < 0.001; ****p < 0.0001. Dot plots show the results of biological replicates, the median (horizontal lines) and the statistical significance (*t*-test).

## SUPPLEMENTARY FIGURES, VIDEOS AND TABLE



## Abbreviations

BrdU: 5-bromo-2′-deoxyuridine
CPT: carboplatin
CPC: cancer propagating cell
EMT: epithelial-mesenchymal transition
FC: fold change
FPKM: fragments per kb per million
IPA: Ingenuity Pathway Analysis
MTT: 3-[4,5-dimethylthiazol-2-yl]-2, 5-diphenyl tetrazolium bromide
PBS: phosphate-buffered saline
RNA-Seq: RNA sequencing
RT-qPCR: reverse transcriptase quantitative PCR
SASP: senescence-associated secretory phenotype.
