# Supplementary material for: A multi-stage process including transient polyploidization and EMT precedes the emergence of chemoresistent ovarian carcinoma cells with a dedifferentiated and pro-inflammatory secretory phenotype
Source: Oncotarget. 2015 Oct 19;6(37):40005–25. doi: 10.18632/oncotarget.5552 (PMC4741876; doi:10.18632/oncotarget.5552)
Supplement: Supplementary file 1 [file oncotarget-06-40005-s001.pdf]

SUPPLEMENTARY FIGURES, VIDEOS AND TABLE

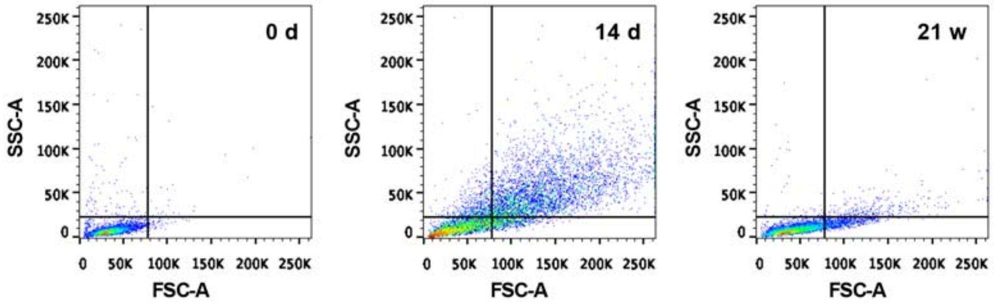

Supplementary Figure S1: Sideward and forward scattering (FACS) of unstained cells after different times of CPT treatment. Forward scatter (FSC-A) correlates with cell size, sideward scatter is an indicator of cellular granularity.

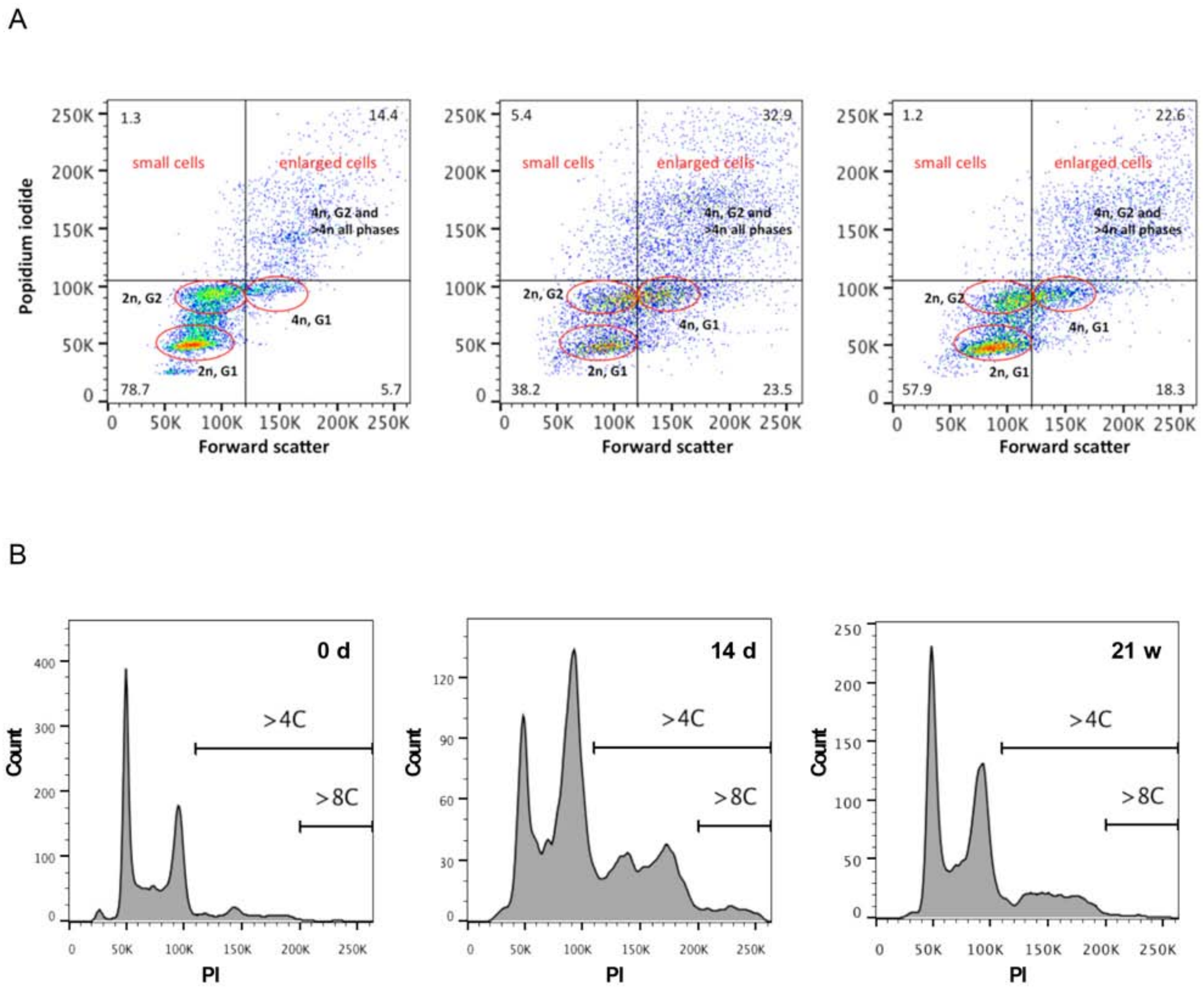

Supplementary Figure S2: FACS dot plot (A) and histogram (B) of propidium iodide (PI) stained cells after different times of CPT treatment. Gates in panel A were set according to the G1and G2 populations on day 0. A 4C DNA content in panel B corresponds to tetraploid cells in G_0/G_1.

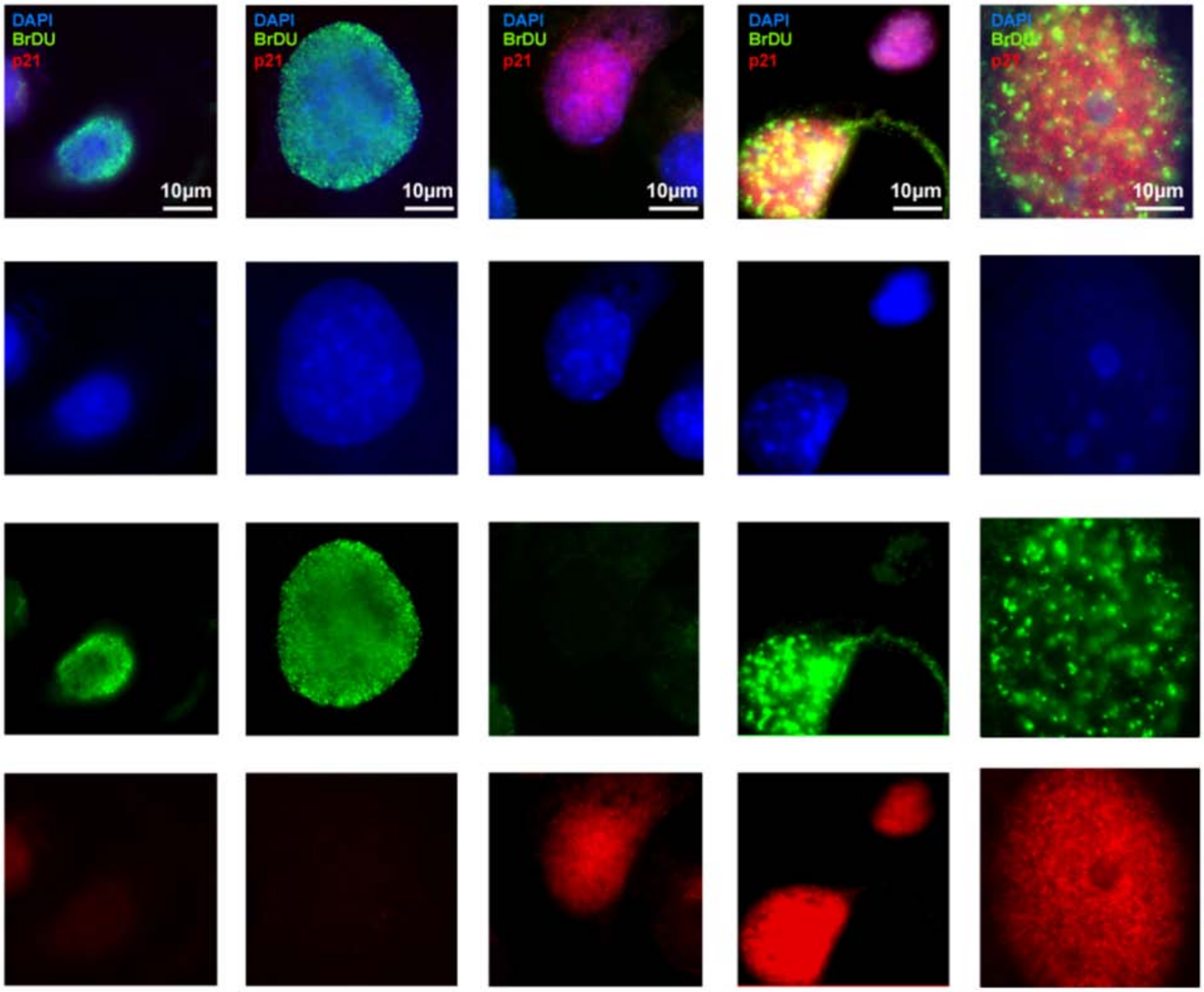

Supplementary Figure S3: Analysis of cell cycle parameters in individual cells after CPT treatment of SKOV3 cells for 14 days. SKOV3 cells were treated with CPT for the indicated times andanalyzed by immunofluorescence (red: p21; green: BrdU) and staining of DNA with DAPI(blue). The top row shows the merged images.

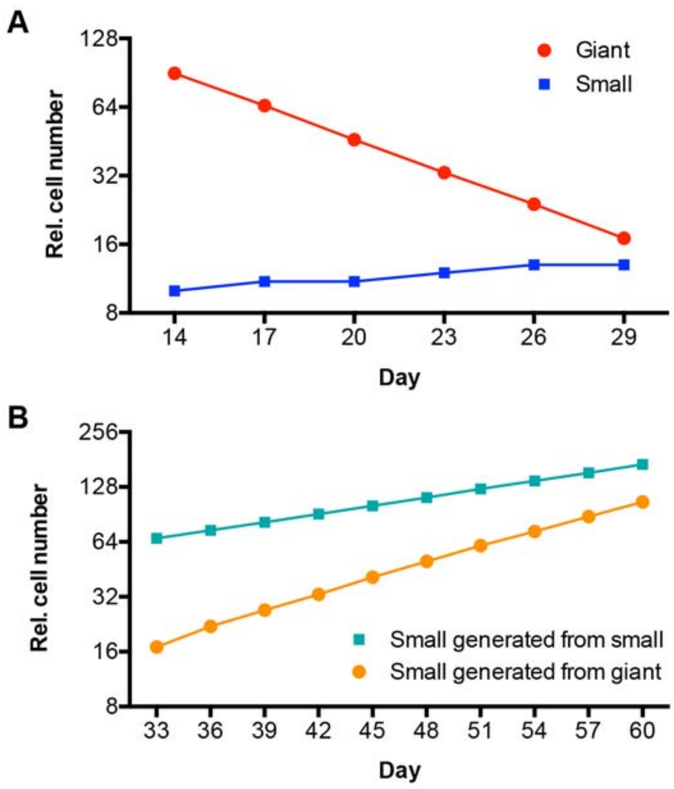

Supplementary Figure S4: Simulation of cell fate under CPT treatment based on life cell imaging data. A. Prediction of small and giant cell numbers between day 14 and 29 (corresponding to 5 cycles starting on day 14). A cycle is defined as a modeled 3-day observation period. **B.** Prediction of numbers of small cells from giant cells or small cells between day 33 and 60 (corresponding to 10 cycles starting on day 30), including small cells generated from giant cells between day 14 and 29). Parameters: cell number measured at the start of simulation period or predicted for the respective preceding cycles (n); fraction of cells dividing into 2 daughter cells (d) or >2 daughter cells (dd); resting cells (r). Values were derived from Figures 1C and 3A as follows: Parameters in panel A for giant cells: n=90; d=0.26; dd=0.010; r=0.16 Parameters in panel A for small cells: n = 10; d = 0.41; dd = ; r = 0.24 Parameters in panel B for giant cells: n = 40; d = 0.39; dd = 0.015; r = 0.325 Parameters in panel B for small cells: n = 60; d = 0.36; dd = 0; r = 0.39 Average total number of daughter cells generated from one giant cell per observation period estimated from video recordings: m = 3.7 Predicted numbers of small or giant cells (k1) are based on iterative calculations using the following formula: k = n•d•2 + n•dd•m + n•r. In B, k was determined as above and used to predict the numbers of small cells derived from or giant or small cells present at the start of the simulation (day 30) using the same mathematical approach.

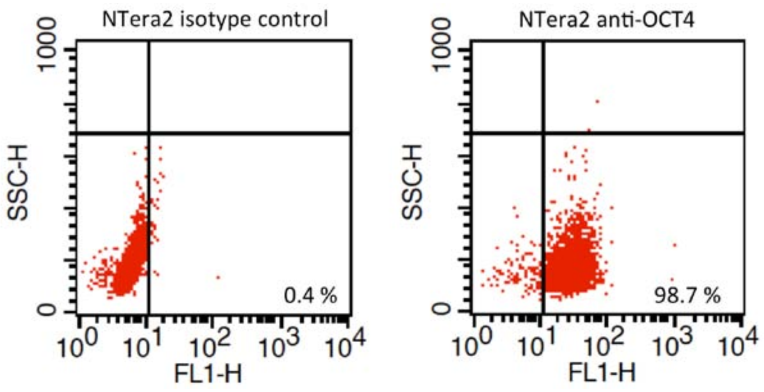

Supplementary Figure S5: FACS dot plot of NTera cells stained with isotype control and anti-OCT4 antibodies. NTera is a human embryonic carcinoma cell line displaying stem cell features, including high OCT4 expression (Pal et al., 2006), and were used to validate the anti-OCT4 antibodies used in the present study. The data show that 98.7% of the cells were specifically stained by the OCT4 antibody. In contrast, <12%% of SKOV3 cells stained positive for OCT4 under the same conditions (see Figure 5A, left panel).

REFERENCE

1. Pal R, Ravindran G (2006) Assessment of pluripotency and multilineage differentiation potential of NTERA-2 cells as a model for studying human embryonic stem cells. Cell Prolif 39: 585–598.

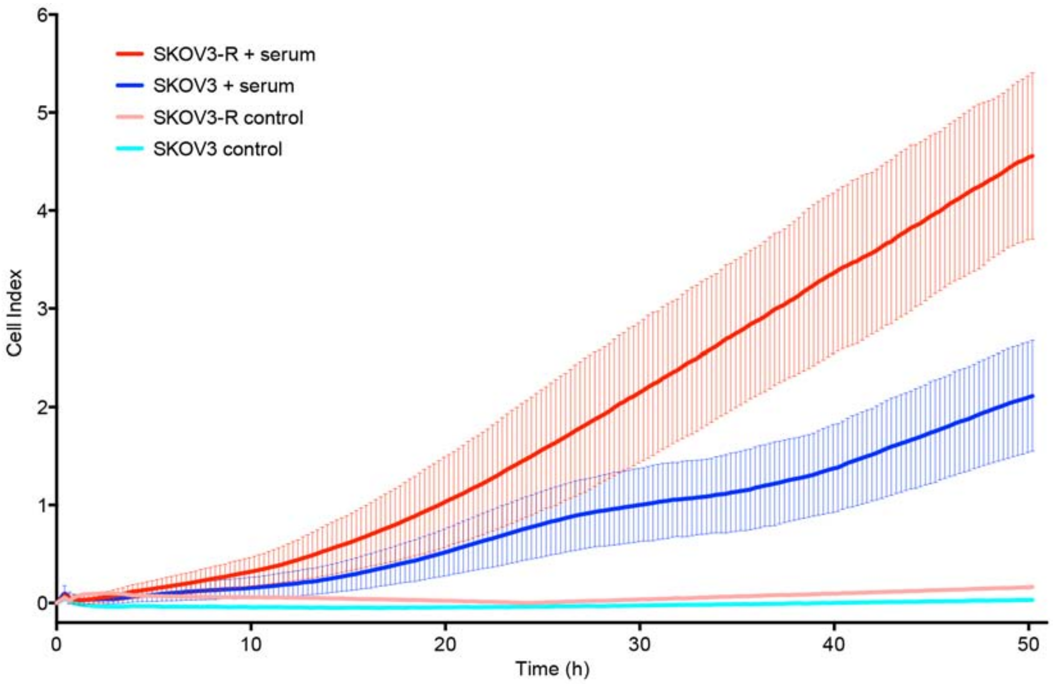

Supplementary Figure S6: Directional migration of SKOV-R versus parental SKOV3 cells. – Quantification of cell migration towards a source of fetal calf serum (FCS) compared to control cells (no serum) was performed by electrical impedance measurements using the xCELLIGENCE real-time cell analyzer (ACEA Biosciences, San Diego, Ca) as described (Jurmeister et al., 2012; Knopfova et al., 2012). The principle of this assay is to monitor the migration of cells through a membrane into the bottom chamber in response to FCS as a chemoattractant, where they adhere to sensors and thereby cause an increase in electrical impedance, which correlates with the cell number. These changes in electrical impedance are used to calculate a "cell index" as an indirect measurand of the number of cells arriving at the senor. The data shown are derived from three independent experiments, error bars indicate the standard deviations.

REFERENCES

1. Jurmeister S, Baumann M, Balwierz A, Keklikoglou I, Ward A, Uhlmann S, Zhang JD, Wiemann S, Sahin O (2012) MicroRNA-200c represses migration and invasion of breast cancer cells by targeting actinregulatory proteins FHOD1 and PPM1F. Mol Cell Biol 32: 633–651

2. Knopfova L, Benes P, Pekarcikova L, Hermanova M, Masarik M, Pernicova Z, Soucek K, Smarda J (2012) c-Myb regulates matrix metalloproteinases 1/9, and cathepsin D: implications for matrix-dependent breast cancer cell invasion and metastasis. Mol Cancer 11: 15.

	0 d	14 d	21 w
Size nucleus (µm²)	1,0	7,4	1,5
Size whole cell (µm²)	1,0	10,9	1,3
BrdU⁺	1,7	1,0	1,5
> 8C	1,0	18,7	7,9
> 4C	1,0	4,8	2,1
β-gal⁺	1,0	31,2	106,5
BrdU⁻p21⁺	1,0	193,5	15,3
p21⁺	1,0	43,8	6,1
CDKN1A	1,0	27,7	3,7
IL8	1,0	244,8	11,3
IL1b	1,0	1015,5	91,2
CCL20	1,0	134,3	3,5
CXCL1	1,0	2,7	1,8
SERPINE1	1,0	12,0	12,3
OCT4 MFI	1,0	1,9	2,3
CD117 MFI	1,0	2,1	1,3
CD24 MFI	1,0	1,3	1,3
CD44 MFI	1,0	2,6	2,0
CD133 MFI	1,0	1,7	1,3
NANOG MFI	1,1	1,8	1,0
SOX2 MFI	1,0	1,3	1,0
CDH1	50,0	7,1	1,0
ACTA2	1,0	2,0	2,6
SNAI2	1,0	4,4	5,0
SNAI1	1,0	8,5	0,9

<1,5
1,5-5
5-10
10-50
>50

Supplementary Figure S7: Temporal occurrence of events under CPT treatment. The heat map shows the highest values in deep red (> 50-fold), the lowest in yellow (<1.5-fold) and intermediate values in three shades of orange.

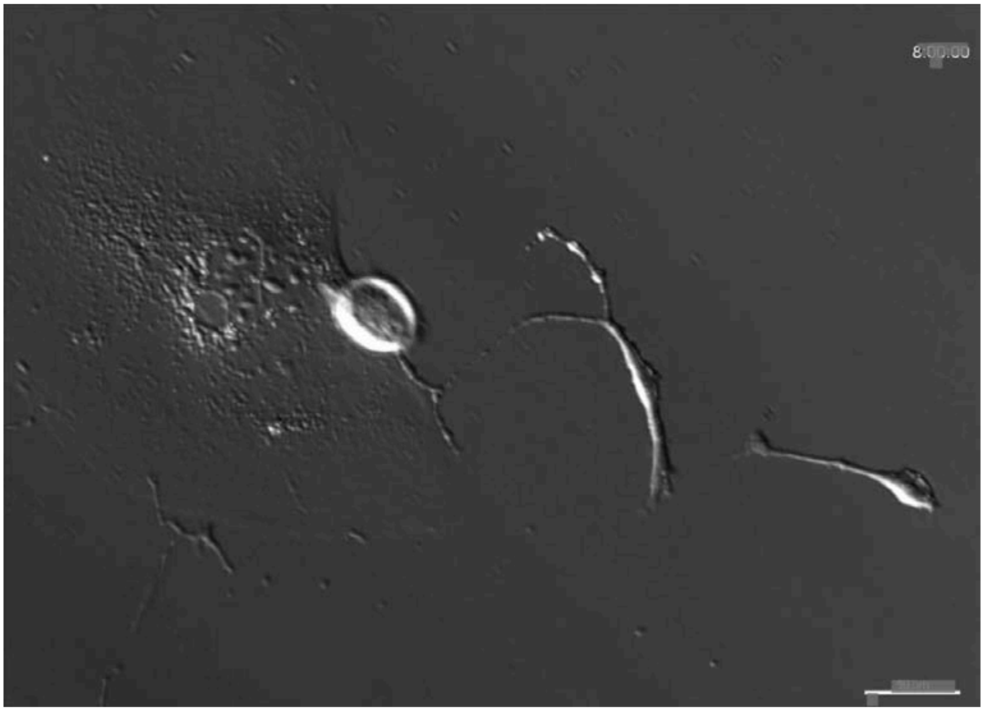

Supplementary Video S1

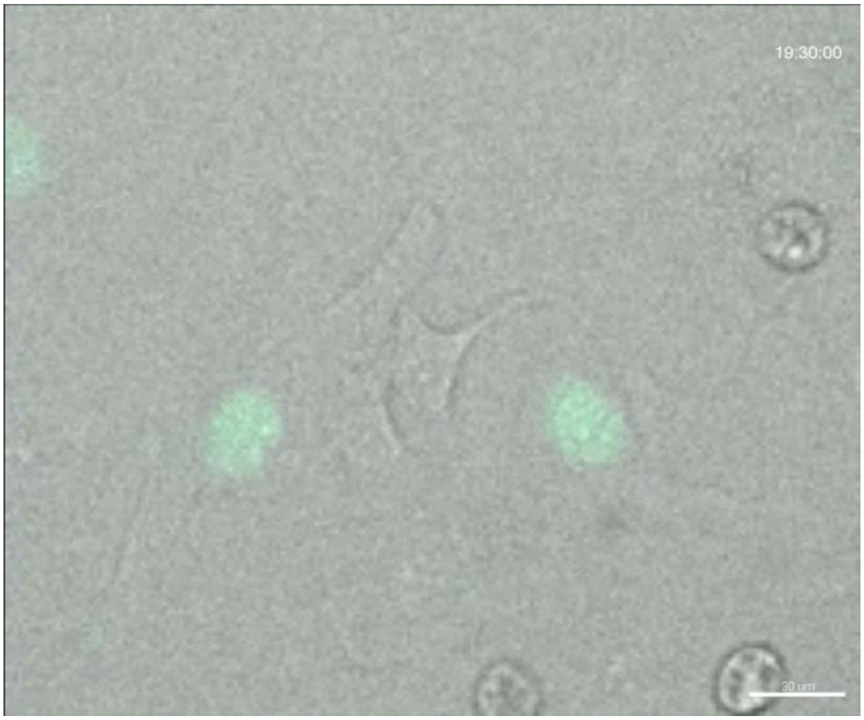

Supplementary Video S2

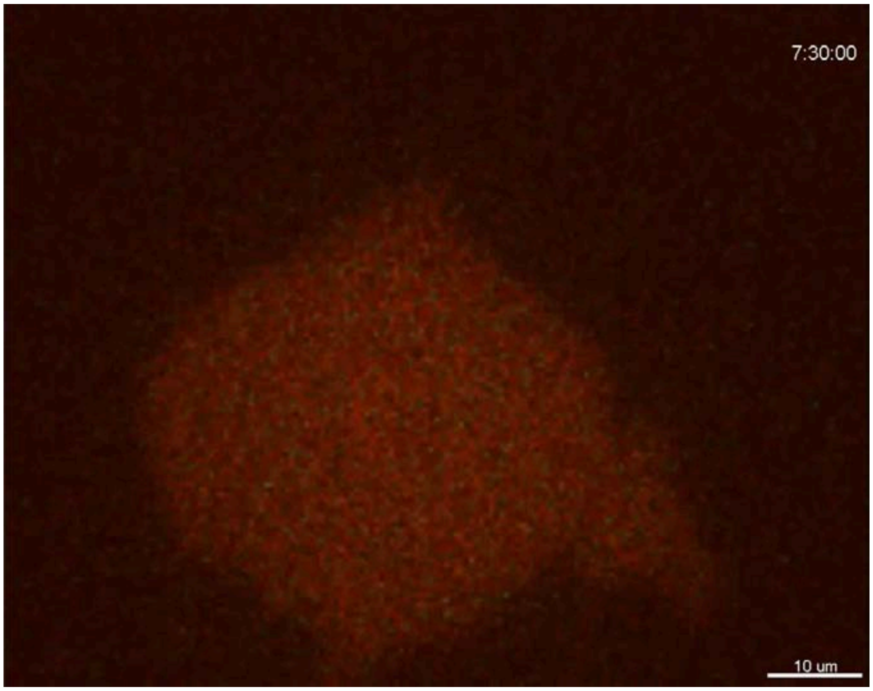

Supplementary Video S3

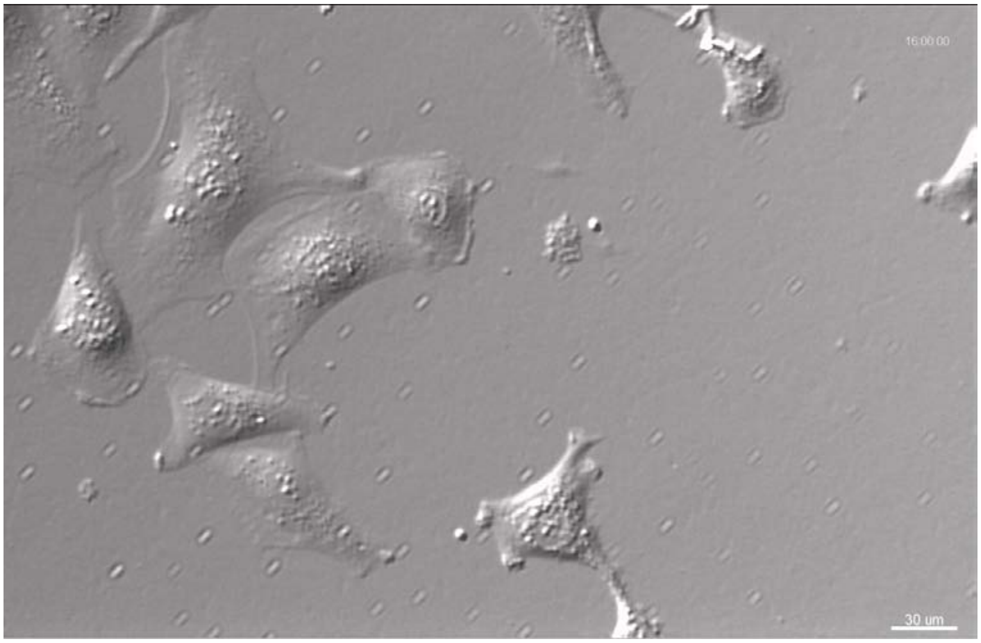

Supplementary Video S4

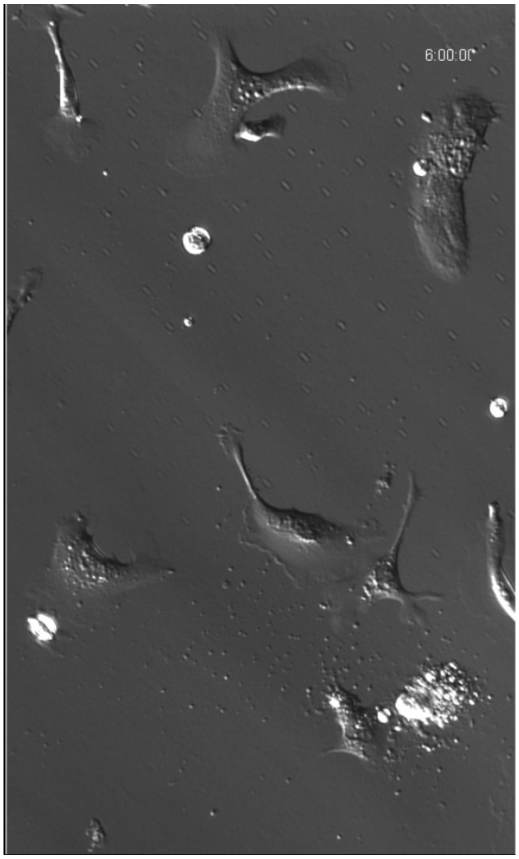

Supplementary Video S5

Supplementary Table S1: Primers used for RT-qPCR-analyses

name	sequence
ACTA2_fw	5' CTGTTCCAGCCATCCTTCAT
ACTA2_rv	5' TCATGATGCTGTTGTAGGTGGT
CCL20_fw	5' GCTGCTTTGATGTCAGTGCT
CCL20_rv	5' GCAGTCAAAGTTGCTTGCTTC
CDH1_fw	5' AGAGCCCTTACTGCCCCCAGAGGAT
CDH1_rv	5' CCTGTGCAGCTGGCTCAAGTCAAAG
CDK6_fw	5' TGATCAACTAGGAAAAATCTTGGA
CDK6_rv	5' GGCAACATCTCTAGGCCAGT
CDKN1A_fw	5' GGCAGACCAGCATGACAGATT
CDKN1A_rv	5' GCGGATTAGGGCTTCCTCTT
CTGF_fw	5' TGCGAAGCTGACCTGGAAGAGAACA
CTGF_rv	5' TCGGCCGTCGGTACATACTCCACA
CXCL1_fw	5' CGAAAAGATGCTGAACAGTGA
CXCL1_rv	5' GCCTCTGCAGCTGTGTCTC
CYP24A1_fw	5' AACTCCCCATCGCGTTTT
CYP24A1_rv	5' AGCAGTGAACCCTGTAGAATG
CYR61_fw	5' AGTGCCGCCTTGTGAAAG
CYR61_rv	5' TGGTCTTGCTGCATTTCTTG
EPCAM_fw	5' AGTCGTCGCTGGAATTGTTGTG
EPCAM_rv	5' GCCTTCTCATACTTTGCCATTC
ERBB2_fw	5' AGGAGGCCTGCGGGAG
ERBB2_rv	5' TTCCGCTGGATCAAGACCCC
FOXP1_fw	5' TCTATGGACATGGTGTATGCAAG
FOXP1_rv	5' CGCATGCTCACTGTTGAGA
IL1B_fw	5' TGAAAGCTCTCCACCTCCAGGGACA
IL1B_rv	5' GAGGCCCAAGGCCACAGGTATTTTG
IL6_fw	5' AGGAACAAGCCAGAGCTGTGCAGATG
IL6_rv	5' TTTGTGGTTGGGTCAGGGGTGGTTA
IL8_fw	5' ATTTCTGCAGCTCTGTGTGAAG
IL8_rv	5' GATAAATTTGGGGTGGAAAGGT
KITLG_fw	5' GCATTGCCAGCATTGTTTTCTC
KITLG_rv	5' GCCCTTGTAAGACTTGGCTG
KRT5_fw	5' AGAAGGCCAAGCAGGACAT
KRT5_rv	5' TGGTCCAACTCCTTCTCCAC
KRT7_fw	5' CAGGCTGAGATCGACAACATC
KRT7_rv	5' CTTGGCACGAGCATCCTT

(*Continued*)

name	sequence
KRT81_fw	5' GCTATGTGAAGGCATTGGGGC
KRT81_rv	5' GTCCCCGCACACGACC
L27_fw	5' AAAGCTGTCATCGTGAAGAAC
L27_rv	5' GCTGTCACTTTGCGGGGGTAG
MT2A_fw	5' CAACCTGTCCCGACTCTAGC
MT2A_rv	5' CATTTGCACTCTTTGCATTTG
MUC16_fw	5' GGCTGCAGACTGACCTCTCT
MUC16_rv	5' TTTTGGGGTCAAGATGATGG
POU5F1_fw	5' GACAACAATGAAAATCTTCAGGAGA
POU5F1_rv	5' TTCTGGCGCCGGTTACAGAACCA
SERPINE1_fw	5' CACAAATCAGACGGCAGCACT
SERPINE1_rv	5' CATCGGGCGTGGTGAACTC
SNAI1_fw	5' CCTTCTCTAGGCCCTGGCTGCTACAA
SNAI1_rv	5' GCACACGCCTGGCACTGGTACTTCT
SNAI2_fw	5' CCAGACCCTGGTTGCTTCAAGGACA
SNAI2_rv	5' TGCTCTGTTGCAGTGAGGGCAAGAA
SRGN_fw	5' CCCTCATCCTGGTTCTGGAAT
SRGN_rv	5' TTACTGTCTGGATTGCAGCG
TGFB2_fw	5' TCAGACACTCAGCACAGCAG
TGFB2_rv	5' GCAGCAAGGAGAAGCAGATG
TGFBI_fw	5' CGGCTAAAGTCTCTCCAAGGT
TGFBI_rv	5' TCCTTGTTGACACTCACCACA
THBS1_fw	5' TCTCTGACCTGAAATACGAATGTAG
THBS1_rv	5' AAGGAAGCCAAGGAGAAGTG
VIM_fw	5' GTTTCCCCTAAACCGCTAGG
VIM_rv	5' AGCGAGAGTGGCAGAGGA
